# Operative Preventive Oral Hygiene Protocols in Pediatric Patients with Leukemia: A Non-Randomized Clinical Trial

**DOI:** 10.3390/dj13040164

**Published:** 2025-04-11

**Authors:** Guido Galbiati, Lucia Giannini, Daniela del Rosso, Maria Grazia Cagetti, Cinzia Maspero

**Affiliations:** 1 Dipartimento di Scienze Biomediche, Chirurgiche e Odontoiatriche, Università degli Studi di Milano, 20122 Milan, Italy; guido.galbiati@unimi.it (G.G.); maria.cagetti@unimi.it (M.G.C.); cinzia.maspero@unimi.it (C.M.); 2 Fondazione IRCCS Cà Granda Ospedale Maggiore Policlinico, 20122 Milan, Italy; 3 Dipartimento di Medicina e Innovazione Tecnologica, Corso di Laurea in Odontoiatria e Protesi Dentaria, 21100 Varese, Italy; ddelrosso@studenti.uninsubria.it

**Keywords:** periodontal status, leukemia, pediatric dentist

## Abstract

**Objective:** The objective of this study is to highlight the critical role of pediatric dentists in promoting oral health prevention among leukemia patients. In fact, oral manifestations frequently serve as the initial clinical indicators of leukemia, occurring in up to 10% of cases. In acute myelomonocytic leukemia, oral lesions are observed in 65–90% of patients. **Methods:** A cohort of 63 patients (30 males and 33 females) with a mean age of 10 years participated in this study. All participants adhered to a standardized preventive dental care protocol, referred to as the “Preventive Iter”. This protocol focuses on individualized oral hygiene education, regular monitoring, and professional dental interventions aimed at preventing and managing oral health complications associated with systemic conditions such as leukemia. **Results:** The results demonstrated a statistically significant and progressive improvement across all evaluated oral health parameters as patients advanced through the Preventive Iter protocol. **Conclusions:** The Preventive Iter protocol has proven to be highly effective in improving oral health outcomes, as evidenced by notable reductions in plaque accumulation, microbial imbalance, and gingival inflammation. The structured, individualized approach—incorporating professional hygiene interventions and tailored educational strategies—appears to be a key factor in achieving and maintaining these improvements. These findings underscore the critical importance of early and ongoing preventive care, particularly for medically vulnerable populations.

## 1. Introduction

Leukemia, also referred to as leucosis, is a group of hematologic malignancies characterized by the uncontrolled proliferation of leukocytes. These malignant cells predominantly proliferate within the bone marrow, spleen, and lymph nodes, and can infiltrate other organs, leading to systemic complications. Although leukemia does not form a distinct tumor mass, it is classified as a neoplastic disease. Over the past century, advancements in therapeutic strategies have significantly improved survival rates. In the early 20th century, 95% of leukemia patients succumbed to the disease; however, modern treatments have improved survival, with approximately 60% of treated individuals recovering [1].

This improvement reflects a paradigm shift in healthcare, emphasizing prevention, public education, and early diagnosis. These principles are particularly pertinent to dentistry, where the early detection and management of oral diseases can significantly enhance patient outcomes. Oral health is of particular importance in leukemia patients due to the complex interactions between the disease, its treatments, and associated oral manifestations [2,3].

Acute myeloid leukemia (AML), the most common form of leukemia in adults, is treated with regimens including chemotherapy, hematopoietic stem cell transplantation, and immunosuppressive agents. However, these treatments often lead to complications such as cytopenia, infections, gingival hyperplasia, oral ulcerations, and altered bone metabolism, all of which present significant challenges for dental management. In pediatric populations, leukemia accounts for approximately 25% of all cancers, with acute lymphoblastic leukemia (ALL) being the most prevalent subtype in children [4,5,6,7].

Oral manifestations frequently serve as the initial clinical indicators of leukemia, occurring in up to 10% of cases. In acute myelomonocytic leukemia, oral lesions are observed in 65–90% of patients. These manifestations, including gingival enlargement, mucositis, oral bleeding, and altered taste, may arise from the disease itself, its treatment, or a combination of both. Understanding these oral complications is crucial for tailoring dental care and minimizing adverse outcomes [4,5].

A multidisciplinary approach is essential for managing oral health in leukemia patients [1,2,3,4,5,6,7,8]. Dental professionals, particularly pediatric dentists, play a crucial role in mitigating oral complications and improving the quality of life for these patients. To address the specific needs of leukemia patients, the “Preventive Iter” protocol was developed. This standardized six-step procedure, initially established in the 1980s at the University of Milan’s Orthodontics Department, emphasizes personalized oral hygiene education and continuous monitoring [8,9,10].

This preventive protocol permits us to achieve great results and a high number of successes, founded on both a patient’s attitude and collaboration.

The prevention protocols have been studied and used since 1985 to educate and motivate patients to achieve and maintain domiciliary oral hygiene care. Although it was introduced more than 40 years ago, it still proves to be very effective.

The protocol incorporates comprehensive oral examinations, plaque and bleeding index assessments, professional hygiene sessions, and fluoride topical applications, when indicated.

It is important to inform the patient about detailed oral hygiene instructions, including periodic scaling and polishing. The preferred brushing technique is the modified Bass technique. All the patients are required to complete of a food diary that should be checked periodically in order to make corrections and changes.

Modifications are made to accommodate the specific needs of leukemia patients undergoing chemotherapy or radiotherapy, ensuring effective oral health management while maintaining patient cooperation. This multidisciplinary approach, coupled with psychological support, fosters a holistic care framework that not only addresses oral health but also enhances overall well-being.

This paper aims to emphasize the critical role of pediatric dentists in promoting oral health prevention among leukemia patients. By implementing specialized professional and home-based oral hygiene protocols, the risk of secondary infections affecting oral tissues can be minimized, thereby contributing to improved patient outcomes and quality of life. The results of this study will evaluate the impact of the Preventive Iter protocol on oral health outcomes, with a particular focus on reducing plaque accumulation, improving gingival health, and managing microbial composition in the oral cavity.

The null hypothesis of this study is that there is no significant difference in the oral health parameters before and after the ‘Preventive Iter’ protocol in leukemia patients.

## 2. Materials and Methods

This study involved a cohort of 63 patients (30 males and 33 females; 48% of the participants were male, and 52% were female) with a mean age of 10 years. The patients who provided consent were included in this study, and these make up approximately 90% of those contacted. All participants were enrolled following a standardized preventive dental care protocol, “Preventive Process”. This protocol emphasizes individualized oral hygiene education, regular monitoring, and professional dental care to manage and prevent oral health complications associated with systemic conditions such as leukemia. The Prevention Program is divided into four stages, in which specific and standardized procedures are carried out on the patient; checkups are then planned after 4 months.

### 2.1. Inclusion Criteria

- Patients diagnosed with leukemia, receiving treatment at the time of this study.
- Patients who provided written informed consent.
- Patients with no severe oral health complications that could interfere with the evaluation of the protocol.
- Patients who were willing to adhere to the oral hygiene protocol for the duration of this study.

### 2.2. Exclusion Criteria

- Patients with severe systemic conditions or other immunocompromising diseases unrelated to leukemia that could affect oral health outcomes.
- Patients with a history of major oral surgery or dental treatments.
- Patients who were unable to comply with the study protocol, including follow-up appointments and oral hygiene requirements.

Dentists should be aware of possible oral manifestations because they have an important role to play before pediatric leukemia diagnosis and during children’s follow-up to ensure adequate oral healthcare. This can help with early diagnosis and a better quality of life during and after leukemia treatment. For this reason, each patient underwent a series of evaluations and interventions as part of the protocol.

To assess the progression and effectiveness of the preventive measures, oral samples were collected at four distinct time points:

At baseline (T0), after the first phase of intervention (T1), following continued monitoring (T2), and at the conclusion of the study period (T3).

At the end of the protocol, check-ups are planned every 4 months.

At each time point, the following parameters were evaluated:

1. Plaque Index (PI): Assessed using the Silness–Löe method to quantify plaque accumulation [8].
2. Bleeding Index (BI): Measured to evaluate gingival health and the presence of inflammation [8].
3. Microbial analysis: The oral samples were examined microscopically to quantify the presence of cocci and bacilli, and further classified into Gram-positive and Gram-negative bacteria.

We used a software program (IBM SPSS Software Statistics version 28) to calculate and analyze the proportions of cocci and bacilli in the dental plaque samples.

Statistical analyses were performed using paired *t*-tests and repeated measures ANOVA.

Professional dental hygiene procedures, including plaque and tartar removal, fluoride applications, and oral hygiene instructions, were provided at each interval to ensure optimal oral health. Patients who exhibited elevated plaque or bleeding indices or unfavorable microbial profiles were given additional guidance and repeated instructions to enhance their oral hygiene practices. The clinical evaluations have been performed by the same practitioner with decades of experience in the field.

As a control group, we used data from our previous studies [9].

To calculate the sample size, we used the sample size calculator software (https://www.epicentro.iss.it/strumenti/samplesize, accessed on 2 March 2023) offered by the Italian Higher Institute of Health. The required sample size for this study was calculated using a statistical formula based on a significance level of 0.05, a power of 90%, and an expected effect size of 0.20, which is a moderate difference between the groups. This calculation suggested a sample size of 94 participants. However, our study includes 63 participants, which is still considered a reasonable number for detecting meaningful differences in the outcomes. Despite being slightly smaller than the calculated ideal, this sample size provides sufficient power to draw valuable conclusions. Future studies will aim to increase the sample size to improve statistical power and further validate the findings.

## 3. Results

The results demonstrated a statistically significant and progressive improvement in all the evaluated oral health parameters as patients advanced through the Preventive Process protocol.

**Figure 1:**
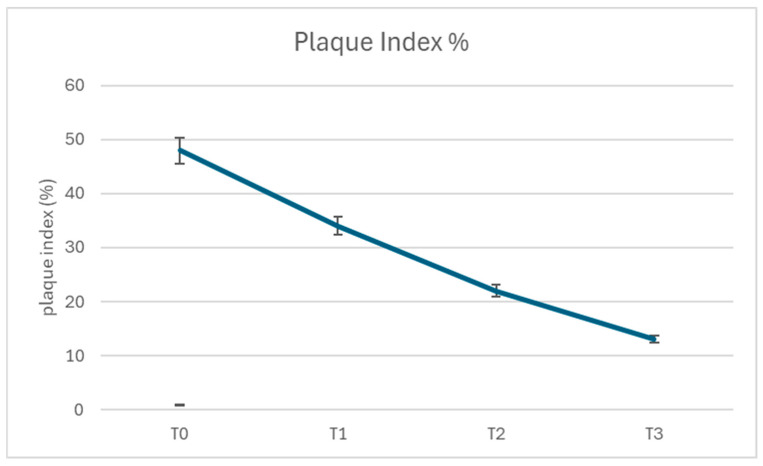
Plaque Index through the protocol.

At baseline (T0), the Plaque Index (PI) had a mean value of 48% (±5.2%). Over the course of this study, PI values decreased significantly at each subsequent time point, with mean values of 34% (±4.8%) at T1, 22% (±3.9%) at T2, and 13% (±3.1%) at T3 (*p* < 0.001 for all comparisons between consecutive time points). The trend indicates a continuous and statistically significant reduction in plaque accumulation as patients progressed through the protocol (Figure 1).

**Figure 2:**
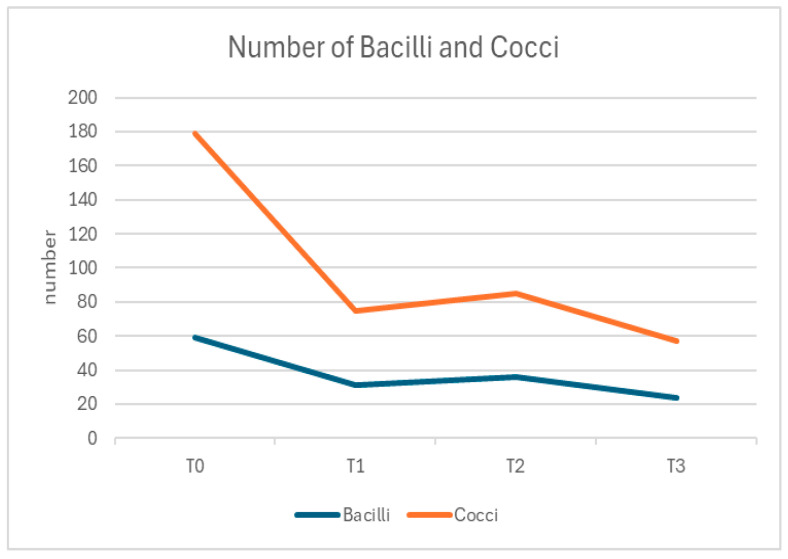
Number of Bacilli and Cocci through the protocol.

**Figure 3:**
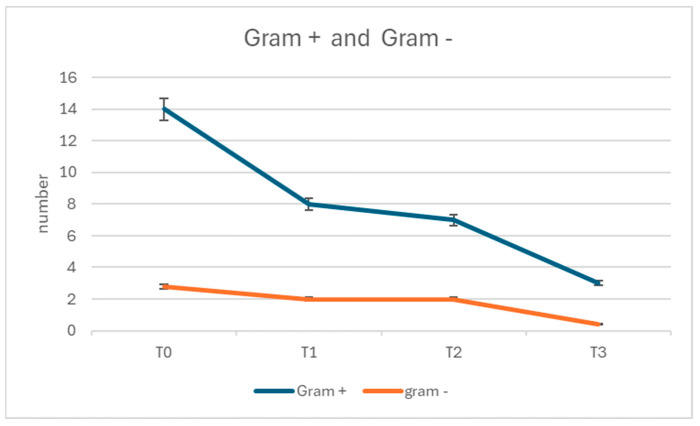
Gram + and Gram - through the protocol.

The microbial composition also showed a significant improvement. At T0, the mean proportion of cocci and bacilli was 70% (±6.5%) and 30% (±4.2%), respectively, with Gram-positive bacteria accounting for the majority of the microbial load (85% ± 5.8%). Over time, these proportions shifted, with significant reductions in total microbial counts and a balanced microbial profile by T3. At T3, cocci accounted for 45% (±4.3%) and bacilli for 15% (±3.6%) of the microbial load, with a marked reduction in Gram-negative bacteria (*p* < 0.001 for changes in microbial composition) (Figure 2 and Figure 3).

**Figure 4:**
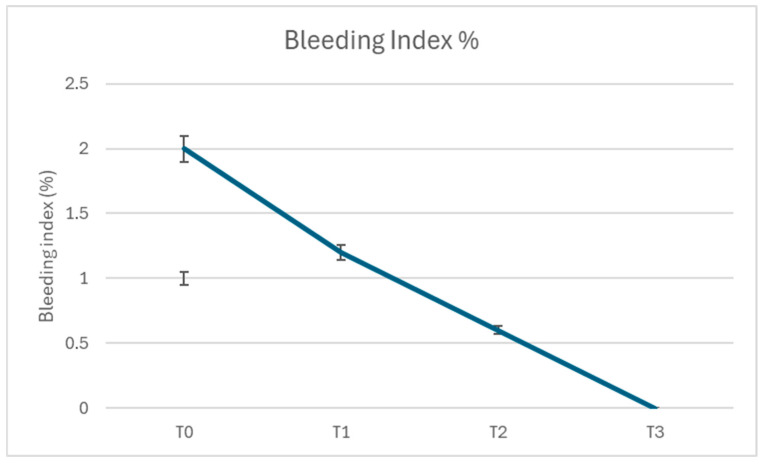
Bleeding Index % through the protocol.

Similarly, the Bleeding Index (BI) exhibited a notable decline. At T0, the mean BI was 2.0 (±0.3), indicating significant gingival inflammation and bleeding. This value decreased to 1.2 (±0.2) at T1, 0.6 (±0.2) at T2, and reached 0.0 (±0.1) at T3 (*p* < 0.001 for all comparisons) (Figure 4). The progressive reduction in BI highlights the resolution of gingival inflammation over the course of this study.

Statistical analyses, performed using paired *t*-tests and repeated measures ANOVA, confirmed the significance of the observed improvements across all time points (*p* < 0.001).

The significant and consistent improvements in plaque control, microbial reduction, and gingival health observed in this study underscore the efficacy of the Preventive Process protocol. These results highlight the importance of structured oral hygiene interventions in maintaining optimal oral health, particularly in medically vulnerable populations.

## 4. Discussion

The results of this study underscore the efficacy of the Preventive Process protocol in significantly improving oral health parameters over time [3,8]. A consistent decline in plaque accumulation, microbial load, and gingival inflammation suggests that a structured and personalized preventive strategy can substantially enhance oral hygiene outcomes, particularly in medically vulnerable individuals.

Regarding the null hypothesis, the results demonstrated a significant difference, with improvements observed in all evaluated oral health parameters following the completion of the preventive protocol.

The significant reduction in the Plaque Index (PI) at all time points emphasizes the importance of regular professional hygiene treatments combined with tailored oral hygiene education. These findings align with prior research highlighting the crucial role of routine professional care and customized oral hygiene instruction in maintaining effective plaque control. The continuous reduction in PI further suggests that reinforcing good hygiene practices over time leads to sustained improvements in oral cleanliness [11,12].

Microbial analysis also supports the effectiveness of the Preventive Process protocol. The initial dominance of cocci and the imbalance between Gram-positive and Gram-negative bacteria emphasize the vulnerability of the oral microbiome to inadequate hygiene. By T3, the shift toward a more balanced microbial composition mirrors previous studies that demonstrate how structured hygiene interventions can positively alter the microbial profile, reducing pathogenic bacterial loads [13]. The notable reduction in Gram-negative bacteria is particularly important, as these species are often linked to periodontal inflammation and disease progression [2,14]. These results suggest that a structured hygiene regimen not only controls plaque but also fosters a healthier microbial equilibrium, which is essential for preventing long-term oral diseases.

Similarly, the Bleeding Index (BI) showed a marked reduction throughout this study, reaching minimal levels by T3. This decline signifies a significant decrease in gingival inflammation and reinforces the role of the Preventive Process protocol in maintaining gingival health. These findings are consistent with prior research that emphasizes the importance of consistent oral hygiene education and mechanical plaque removal in minimizing gingival bleeding and inflammation [15]. The substantial improvements in BI highlight the critical importance of early and continued preventive care in managing gum health, particularly for individuals prone to inflammatory conditions.

Another important observation is the lack of statistically significant differences between males and females in PI, microbial composition, and BI trends. This suggests that the effectiveness of the protocol is not gender-dependent, highlighting the potential for broad applicability across diverse demographic groups without requiring significant modifications. The versatility of the Preventive Process protocol may support its implementation as a standard preventive measure in various populations.

Despite these promising findings, some limitations should be acknowledged. The study cohort, while representative of the target population, is relatively small, and extended follow-up would be valuable for evaluating the long-term impact of the protocol. Additionally, incorporating a control group that does not receive the Preventive Process protocol would allow for a direct comparison with conventional preventive care approaches, further validating these results. Furthermore, examining the impact of external factors such as dietary habits, medication use, and genetic predisposition could offer a more comprehensive understanding of the protocol’s effectiveness and its potential applications.

Patient adherence to the protocol is another crucial consideration. While professional dental care and education were reinforced throughout this study, ensuring long-term compliance outside the study environment remains a challenge. Future research should explore the role of motivational strategies, digital health tools, and caregiver involvement in maintaining sustained adherence to preventive protocols [16,17].

The Preventive Process was tailored to accommodate the individual needs of each patient, with modifications made based on their cognitive abilities, manual dexterity, and overall health status. For younger or less cooperative patients, parents or caregivers were actively involved in the protocol to ensure adherence and maximize its effectiveness.

In summary, this study provides compelling evidence supporting the efficacy of the Preventive Process protocol in enhancing oral health. However, additional research is needed to refine its application, evaluate its long-term sustainability, and assess its effectiveness in diverse patient populations [18,19,20].

Oral health is of paramount importance in pediatric patients with hematological diseases, as these children are particularly vulnerable to infections, bleeding, and complications arising from medical treatments. Hematological diseases can significantly impact oral health, and conversely, poor oral health can exacerbate the underlying disease.

Key risks and complications associated with leukemia and its treatments include immunosuppression and increased susceptibility to infections. Children undergoing chemotherapy or with compromised immune systems face a higher risk of oral infections, such as oral mucositis, candidiasis, gingivitis, and gum bleeding. Additionally, prolonged therapies, including chemotherapy and corticosteroids, can affect dental development, leading to enamel hypoplasia and altered tooth growth. Xerostomia, induced by medications and oncological therapies, further elevates the risk of tooth decay and infections. Pain and feeding difficulties due to mucositis can impair nutrition and hinder growth [21].

Prevention and effective management strategies, such as regular dental monitoring and oral hygiene education, are essential to mitigate these risks and prevent complications. A multidisciplinary approach, involving collaboration among hematologists, pediatricians, and specialized dentists, is mandatory for providing comprehensive care.

Pediatric dentists must be involved from the diagnosis of hematological diseases to prevent complications and ensure a personalized approach to oral care. Oral health plays a crucial role in managing pediatric patients with hematological diseases, as careful prevention and dental care can reduce the risk of complications, improve the child’s quality of life, and support the success of hematological therapies.

Additionally, intergroup comparisons between males and females showed no statistically significant differences in the trends of PI, microbial composition, or BI, suggesting the protocol’s effectiveness was independent of gender.

A limitation of this study is the relatively small sample size, which may affect the generalizability of the findings. Further research with larger sample sizes is needed to confirm these results and better understand the long-term effects of the ‘Preventive Iter’ protocol in leukemia patients

## 5. Conclusions

The Preventive Process protocol has proven to be highly effective in enhancing oral health outcomes, as evidenced by significant reductions in plaque buildup, microbial imbalance, and gingival inflammation. The structured and individualized approach, integrating professional hygiene interventions and customized education, appears to be a crucial factor in achieving and maintaining these improvements.

These findings emphasize the importance of early and continuous preventive care, particularly for medically vulnerable groups. The protocol’s ability to deliver consistent improvements over different time points suggests its potential as a standardized model for preventive oral healthcare. Future studies should aim to examine long-term adherence and sustainability, as well as compare their effectiveness with alternative preventive strategies.

## Data Availability

The data presented in this study are available upon reasonable request, after the signature of a formal data sharing agreement in anonymous form, from the corresponding author, because they are protected by privacy.
